# Considerations Around the Inclusion of Children and Young People’s Time in Economic Evaluation: Findings from an International Delphi Study

**DOI:** 10.1007/s40273-024-01411-w

**Published:** 2024-08-17

**Authors:** Cameron Morgan, Cam Donaldson, Emily Lancsar, Stavros Petrou, Lazaros Andronis

**Affiliations:** 1 Centre for Health Economics at Warwick, Warwick Medical School, University of Warwick, Coventry, CV4 7AL UK; 2 Yunus Centre for Social Business and Health, Glasgow Caledonian University, Glasgow, UK; 3 Department of Health Services Research and Policy, Australian National University, Canberra, Australia; 4 Nuffield Department of Primary Care Health Sciences, University of Oxford, Oxford, UK

## Abstract

**Background:**

People’s time is a finite resource and a valuable input that ought to be considered in economic evaluations taking a broad, societal perspective. Yet, evaluations of interventions focusing on children and young people (CYP) rarely account for the opportunity cost of time in this population. As a key reason for this, health economists have pointed to uncertainty around when it is appropriate to include CYP time-related costs in an economic evaluation and highlighted the lack of clear guidance on the topic.

**Methods:**

With this in mind, we carried out a Delphi study to establish a list of relevant considerations for researchers to utilise whilst making decisions about whether and when to include CYP time in their economic evaluations. Delphi panellists were asked to propose and rate a set of possible considerations and provide additional thoughts on their ratings. Ratings were summarised using descriptive statistics, and text comments were interrogated through thematic analysis.

**Findings:**

A total of 73 panellists across 16 countries completed both rounds of a two-round Delphi study. Panellists’ ratings showed that, when thinking about whether to include displaced CYP time in an economic evaluation, it is very important to consider whether: (1) inclusion would be in line with specified perspective(s) (median score: 9), (2) CYP’s time may already be accounted for in other parts of the evaluation (median score: 8), (3) the amount of forgone time is substantial, either in absolute or relative terms (median score: 7) and (4) inclusion of CYP’s time costs would be of interest to decision-makers (median score: 7). Respondents thought that considerations such as (1) whether inclusion would be of interest to the research community (median score: 6), (2) whether CYP’s time displaced by receiving treatment is ‘school’ or ‘play’ time (median score: 5), and (3) whether CYP’s are old enough for their time to be considered valuable (median score: 5) are moderately important. A range of views was offered to support beliefs and ratings, many of which were underpinned by compelling normative questions.

**Supplementary Information:**

The online version contains supplementary material available at 10.1007/s40273-024-01411-w.

## Key Points for Decision Makers

**Table:** 

Patients’ time is a valuable resource that ought to be accounted for in economic evaluations conducted from a societal perspective. However, children and young people’s (CYP) time is rarely included in such economic evaluations, with research pointing to ambiguity and a lack of guidance around when it may be appropriate to do so as a primary reason.
Our Delphi study highlighted the following considerations as particularly important when judging whether CYP time should be included in an economic evaluation: (i) alignment with specified perspective, (ii) there is a substantial amount of forgone time, (iii) CYP time is not accounted for in other parts of the evaluation and (iv) CYP time costs are of particular interest to decision-makers.
Views gathered from panellists also highlighted that, in some cases, interpretation of relevant considerations requires value judgments.

## Introduction

The last 5 decades have seen a growing number of economic evaluations in health care, with such studies now regularly requested and called upon to inform funding decisions about interventions, programmes and treatments offered to adults and children [1, 2]. Economic evaluations that take a societal perspective have been recommended for capturing and reflecting the breadth of costs and benefits that are necessary to guide optimal decision-making [3–5].

In such evaluations, it is important that all relevant resources and costs are considered, including those contributed or borne by individuals. Time is such a resource. Being an input in all forms of health care provision, patients’ time and its associated opportunity cost need to be taken into account in societal-perspective evaluations, especially if the compared alternatives are characterised by differences in the amounts of time devoted by patients [6, 7]. This is particularly the case when a proposed programme of care requires less of a patient’s time compared with current practice (e.g. due to a shorter procedure, quicker examination, reduced waiting time, etc.), and therefore imposes a lower time-related cost to the individual.

Children and young people’s (CYP’s) time (here, defined as individuals under 18 years of age) is also valuable [8]. Thus, if the process of receiving care results in displaced time which could have been spent in other activities (e.g. educational or leisure activities), this time will need to be measured, converted into a cost and included in calculations [4, 6]. Notwithstanding this, there may be situations when a decision to not include CYP’s time may be pragmatic and justifiable [3, 6]. Yet, there is currently no relevant guidance that researchers can refer to when deciding whether, and in which cases, including CYP’s time-related costs in their economic evaluation may be beneficial, superfluous, or even inappropriate. A recent international survey on the topic gathered views from health economists around the inclusion of CYP’s time-related costs in their analyses [9]. Respondents highlighted the lack of insight around when it is appropriate to include CYP’s time-related costs as a key obstacle to accounting for CYP’s time in economic evaluations conducted from a societal perspective, and an important area for further research [10].

With this in mind, we set out to identify a set of considerations that would help researchers make decisions about whether to include CYP time in their economic evaluation using a modified Delphi study. The rest of this article is organised as follows. In the next section, we describe the methods used to develop and conduct the reported Delphi study. This is followed by a summary of findings, with a focus on panellists’ scores regarding the importance of each consideration, and opinions expressed in open-ended questions aiming to explore their views on the topic and the reasoning behind their scores. Drawing on these findings, we highlight key messages, discuss challenges and pinpoint areas for further research.

## Methods

We pursued the objectives of this study using a modified Delphi study design. Delphi studies have been widely used as a means of eliciting views, gathering group opinions, or reaching consensus on a particular question or topic [11–14].

In a Delphi study, panellists are asked to give their opinion on a statement or topic of interest, usually by rating a number of propositions [15]. The exercise takes place over a number of rounds, allowing participants to reflect on their and the rest of the panellists’ previous answers and enabling them to change their views, should they wish to do so [16]. Whilst some guidance exists in relation to the methodological conduct of a Delphi study [12], the technique is adaptable to the needs of a particular research question. Over the last few decades, the method has gained traction in numerous disciplines, including in social and health sciences [17, 18], where it has been used to elicit and ascertain opinions, explore views, identify policy responses and produce guidance [19–24].

### Participants

**Fig. 1:**
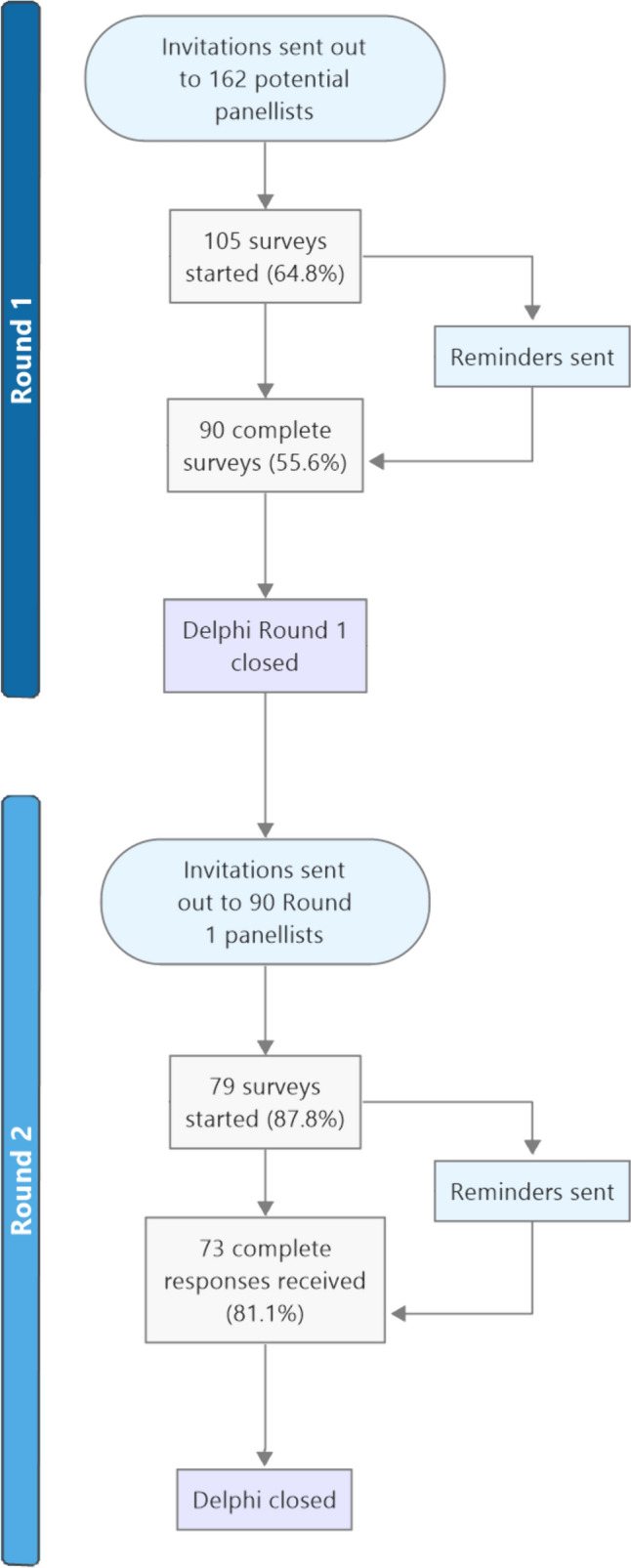
Flowchart of Delphi process

Potential participants were 274 respondents to a recent international survey that aimed to understand health economists’ views in relation to the measurement, valuation and inclusion of CYP’s time in economic evaluations [9]. As part of that survey, respondents were also asked whether they would be interested in being invited to participate in the Delphi study reported here. In total, 162 individuals who completed the preceding international survey expressed an interest in participating in this Delphi study, giving the study’s sampling frame (Fig. 1). Demographic information for panellists who participated in the Delphi study is given in Sect. 3.1 below and, in more detail, in Table 2 of the Supplementary Materials. Due to anonymity requirements, it was not possible to collect demographic information for the individuals who agreed to be invited but opted not to participate.

All participants were assigned a unique ID to enable the tracking of their responses between rounds. Participant identifying information was only accessible to two researchers (C.M. and L.A.). Participants remained anonymous to one another throughout the course of the study.

### Study Design

The Delphi study was guided by a pre-specified protocol agreed upon by all members of the research team. The study was designed to be administered electronically, via the Qualtrics XM survey platform (Qualtrics XM, Provo, UT, USA).

All parts of the study were developed iteratively by two researchers (C.M. and L.A.), with input and feedback provided by the broader research team. Participant-facing documents and forms (e.g. invitation emails, targeted reminders, participant information sheets, consent process and personalised summaries of participant and group’s responses, etc.) were pre-tested with a sample of 15 researchers selected purposefully to match characteristics of the target population. We collected comments by asking respondents to provide written feedback in ‘free-text’ boxes while completing the Delphi study, and we carried out cognitive interviews with five respondents, in the form of ‘think aloud’ sessions with concurrent probing [25]. All cognitive interviews were conducted via teleconferencing and were recorded and transcribed. In addition to providing comments regarding comprehension, possible ambiguity and completeness, pre-testing offered an opportunity to identify potential issues with arrangements related to administration of the Delphi study (e.g. settings for survey distribution and anonymity, retrieval of responses, etc). Aside from cognitive interviews, feedback was provided anonymously, to minimise the chance of acquiescent bias [26]. The project received ethical approval from the University of Warwick Biomedical and Scientific Research Ethics Committee (BSREC 48/22-23).

Key methodological characteristics of the Delphi study, including criteria, decisions, and interpretation of findings, were specified in advance. A table presenting these decisions using the Delphi quality assessment framework recommended by Diamond and colleagues [15] is available as Supplementary Material.

#### Round 1

Invitations to participate in Round 1 of the Delphi study were distributed in March 2023 via e-mail messages containing a link to an electronic questionnaire. Round 1 remained open for 3 weeks; during this period, we provided two targeted reminders to invitees who had not completed the survey.

In the first section of the questionnaire, panellists were presented with a series of questions designed to collect basic demographic information. This included questions related to participants’ experience and familiarity with the conduct and methods associated with economic evaluation, their employment and their country. In the second section, panellists were presented with the question: “While thinking whether to account for CYP time in their economic evaluation, how important is it for a researcher to consider [each of the following options]?” and were asked to rate each of eight considerations on a 9-point scale. Scores denoted the following categories: (1) 1–3 ‘not important’, (2) 4–6 ‘moderately important’ and (3) 7–9 ‘very important’, with an additional option for panellists who were unsure about a given item. In answering these questions, participants were asked to consider CYP as individuals under 18 years of age, and the time in question as an input to an intervention (i.e. time spent being treated, taking part in health promoting activities, etc.), rather than as an outcome of an intervention (i.e. extended survival, quicker return to full health, etc.). Considerations were derived from suggestions in the literature [4, 10], as well as from health economists’ answers to a similar question in a preceding international online survey on the topic [9]. Participants were also asked to suggest additional items, should they feel that important considerations were missing from the provided list. In line with the study’s protocol, new items would be added to the list if they were suggested by at least 10% of respondents.

Targeted, open-ended questions were subsequently presented to participants who selected ‘I’m not sure’ for any of the items, offering an opportunity to provide feedback and identify concerns or confusion with the meaning or phrasing of questions. All respondents were also provided space to offer any general comments or suggestions concerning the Delphi questionnaire.

#### Round 2

Results and feedback obtained from Round 1 were considered whilst planning the second and final round of the Delphi study. Invitations to participate in Round 2 were distributed in May 2023, with two targeted reminders sent before the round closed. Each invitation was accompanied by a document containing information about the panellist’s individual scores, and a table of summary statistics covering Round 1 scores provided by all respondents (mean and median score for each item, and percentage of panellists that rated each item as ‘not important’, ‘moderately important’ or ‘very important’). Respondents were advised that they could disregard or utilise this additional information however they wished.

During Round 2, the panellists were once again asked to score items on the list of considerations. Minor changes were made to the phrasing of some items to reflect feedback received from panellists in Round 1 and during the Round 2 piloting process. No additional items were added on the basis of suggestions received in Round 1, as none met our criteria of being proposed by more than 10% of panellists. This round, too, included a series of open-ended follow-up questions aiming to understand panellists’ reasoning and views. Space was also provided for general comments.

### Analysis

Panellists’ ratings were analysed using descriptive statistics, in line with recommendations [15, 27]. Median scores were calculated for all items rated 1–9, though individuals who selected ‘I’m not sure or I do not know’ were excluded from these calculations. The percentage of responses falling into each category—‘not important’ (scores 1–3), ‘moderately important’ (scores 4–6), ‘very important’ (scores 7–9) and ‘I’m not sure or I do not know’—was also calculated.

Answers to multiple choice and Likert-style questions (i.e. those relating to experience and familiarity with economic evaluation, type of employment, etc.) were analysed descriptively using frequency and percentage tables.

Answers to open-ended (‘free text’) questions were analysed through thematic analysis, with codes and themes developed inductively [28, 29]. After reading through all comments, two researchers (C.M. and L.A.) constructed themes and applied them to the available text. Disagreement arising during the stages of theme development, or discrepancies in the application of code to text, were resolved by discussion and, if necessary (e.g. disagreement persisted), by seeking the views of further members of the project's research team.

Data management tasks were carried out in MS Excel (Microsoft Corp, Redmond, WA, USA), statistical analyses in Stata v17 (StataCorp, College Station, TX, USA) and qualitative data analyses in NVivo 12 (QSR International, Burlington, MA, USA).

## Results

Round 1 of the Delphi study was completed by 90 of the 162 initial invitees (56%). Of them, 73 respondents (81%) went on to complete Round 2 (Fig. 1).

### Respondent Characteristics

Demographic information for Delphi panellists was collected during Round 1 and is provided as Supplementary Information. Constructing a ‘profile’ on the basis of the most commonly reported characteristics, a ‘typical’ respondent was based in the UK (39.7% of all respondents), identified as a health economist (80.8%) and worked in an academic institution (86.3%). Over the 5 years preceding the Delphi, a ‘typical’ respondent had always or almost always been involved in leading or carrying out economic evaluations of health care interventions (41.1%), and they had often carried out methodological research related to economic evaluation (30.1%). The ‘typical’ respondent reported being familiar (79.5%) with the general methods for conducting economic evaluations, and very familiar (82.2%) with the categories of costs included in economic evaluations from different perspectives. They were also familiar (45.2%) with the measurement, valuation and inclusion of individuals’ forgone time in economic evaluation. The ‘typical’ respondent had never been involved in decision-making related to the allocation of health care resources (e.g. as a member of a committee, panel or board making decisions about the adoption of interventions or allocation of research funding; 56.2%).

### Ratings of Considerations

Panellists’ ratings of all items in both rounds of the Delphi can be seen in Table 1. A graph showing Round 2 scores is also provided as Supplementary Information. Scores given for each consideration are examined below, alongside comments received in follow-up questions.

**Table 1.**
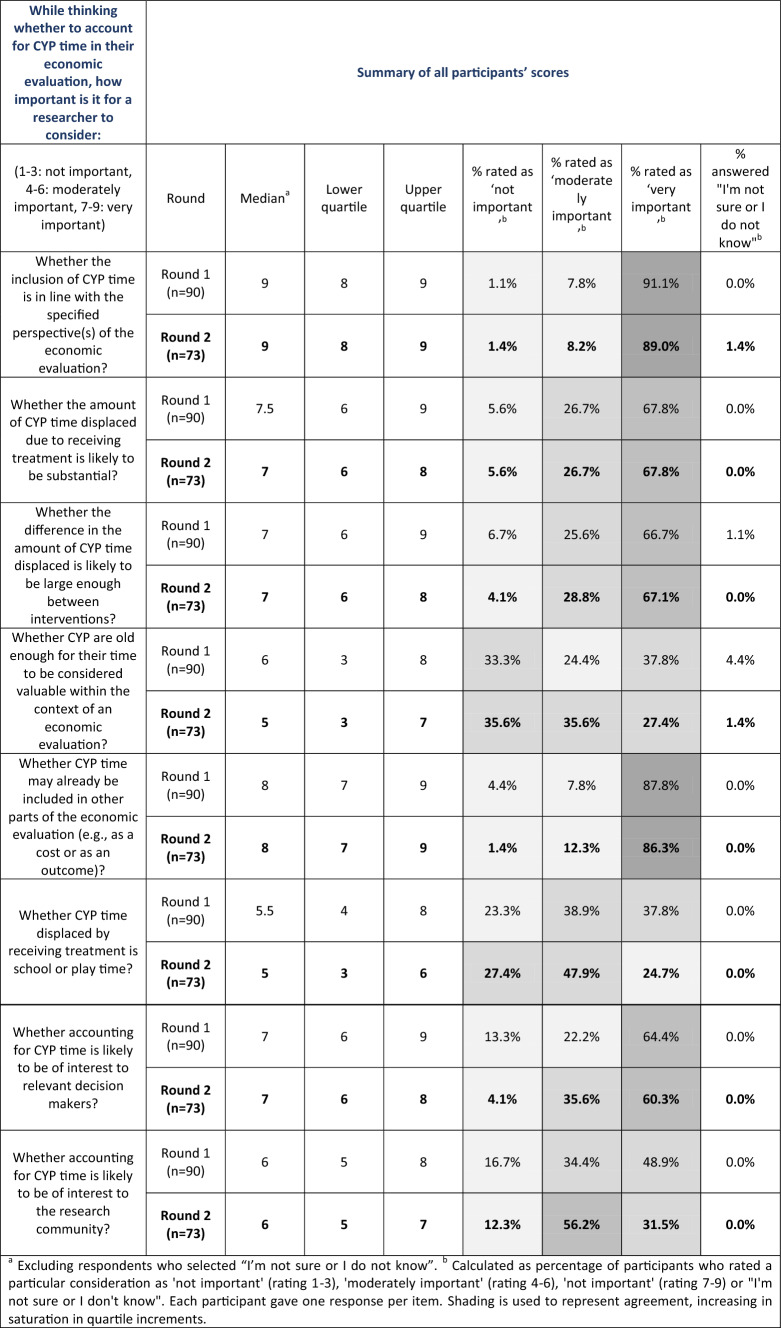
Summary statistics of Delphi ratings in both rounds

^a^Excluding respondents who selected ‘I’m not sure or I do not know’

^b^Calculated as the percentage of participants who rated a particular consideration as ‘not important’ (rating 1–3), ‘moderately important’ (rating 4–6), 'not important' (rating 7–9) or ‘I'm not sure or I don't know’. Each participant gave one response per item. Shading is used to represent agreement, increasing in saturation in quartile increments

#### Consideration 1: Whether Inclusion of CYP Time-Related Costs is in Line with Adopted Perspective

Panellists thought it is moderately important (8%) or very important (89%) that researchers assessing whether they should include CYP’s time in their analysis ought to consider whether this aligns with the specified perspective(s) of the study. The median score for this consideration was 9, placing it at the top end of the ‘very important’ category. Only one panellist rated this consideration as not important, giving it a score of 1. This rating was diametrically opposite to the panellist’s answer during Round 1; thus, it is possible that there was confusion about the directionality of the scale. The spread of scores was limited (lower quartile: 8; upper quartile: 9), with nearly 90% of the scores falling within the category containing the median, demonstrating high levels of agreement between panellists.

Panellists who viewed this consideration as moderately or not important were asked to provide their thoughts behind their ratings. Very few answers were provided to this question, given that almost 90% of panel members rated this consideration as very important. Of those who did provide answers, it was commonly suggested that guidelines or reference cases ought to be expanded to facilitate inclusion of a wider pool of pertinent costs, or that a broader societal perspective may be desirable. Characteristically, a panellist stated: “If CYP time is an important factor, it should be included in the analysis, even if that is not in the primary evaluation, and irrespective of the perspective specified in the primary evaluation.”

#### Considerations 2 and 3: Whether the Absolute or Relative Amount of Forgone Time is Likely to be Substantial

Panellists were asked to rate how important they think it is for a researcher to consider whether the amount of time displaced due to receiving a treatment is likely to be substantial enough to warrant its inclusion, in absolute terms (Consideration 2). Over two-thirds (68%) of panellists viewed this as a very important consideration, and over 23% as moderately important. Fewer than 6% of panel members rated this consideration as not important. There was a high degree of agreement in relation to this item, with a median score of 7 and an interquartile range of 2 (25th percentile: 6; 75th percentile: 8).

A subsequent question asked whether it was important to consider whether the difference between interventions in terms of time displaced is likely to be substantial enough to warrant its inclusion (Consideration 3). Similarly, this was largely viewed as an important consideration, with almost 96% of respondents rating this as moderately or very important. Here, too, there was a high degree of agreement, with a median score of 7 and an interquartile range of 2. As expected, scores for Considerations 2 and 3 were correlated, with most participants attaching the same level of importance to both. Pearson and Spearman correlation analyses gave similar coefficients of approximately 0.52, indicating a positive correlation between scores.

Panellists who rated these considerations as moderately or very important were asked to provide their views on what might be useful for a researcher to think about when judging whether the said quantities are likely to be substantial enough. Several panel members provided their insights or advice about this decision-making process, whilst others provided additional context. The sentiment that, if time displaced, or the difference in time displaced between interventions, is expected to be minimal, the increased expense and complexity of the inclusion of these costs may not be justified was shared by many panellists. However, determining whether the amount of time displaced is likely to be minimal or substantial was recognised as a key challenge, as evidenced by several panel members who highlighted that ‘substantial enough’ is subjective, and it may not be feasible to determine the size of these inputs before it becomes necessary to collect data. As a solution to this, it was noted that clinical input, evidence from the existing literature and meta-analyses may be able to shed some light on the time costs associated with an existing (type of) treatment. However, for new treatments it is plausible that a situation may arise “…when comparing two similar interventions (e.g., two drugs) where a priori is difficult to predict potential adverse events that lead to more CYP time displaced”. Such adverse events may significantly increase patient time costs whilst being difficult to predict in advance.

Others further noted that, alongside health care professionals, patients or their carers may provide useful insight into what should be considered ‘substantial enough’—with one noting “if it is sufficient to register in their minds, it is probably sufficient to be considered ‘substantial’”. A common theme amongst these answers was that the level of disruption caused by an intervention, for instance, comparing treatments utilising a different “mode or place of administration” may help determine whether time should be included, or that priority should be placed on how much of an effect this displaced time has on outcomes (e.g. if treatment results in the missing of education).

Finally, several members of the panel took a more pragmatic approach to the decision regarding ‘substantial enough’—stating that, if the inclusion of CYP’s time has the potential to alter the final decision or recommendation following an economic evaluation, it should be included. In particular, one panellist suggested that “if differences are likely to be 2–5% or more of total incremental cost, then it's an important consideration”.

#### Consideration 4: Whether Time-Related Costs may be Captured in Other Parts of the Evaluation

Consideration of whether CYP’s time may already be included in other parts of the economic evaluation was thought to be important by almost all panellists, with 98.6% rating this item as either moderately or very important. The median score was 8 (lower quartile: 7; upper quartile: 9), and 86.3% of the respondents scored this in the category where the median lies (i.e. ‘very important’), indicating high levels of agreement.

In a follow-up question, panellists were asked to identify any situations in which they felt that CYP time may already be also captured as an additional input (cost) or output (outcome) within an economic evaluation, leading to potential double-counting. On the input side, a number of respondents indicated that they could not think of any situations in which double-counting would arise. However, some panellists suggested it may be considered double-counting if parental or caregiver time is included—for instance, in the form of caregiver time, lost wages, childcare costs or travel expenses. As one panellist noted, however, including these costs “is not double counting as caregiving [opportunity] costs can be considered independently”.

On the output side, some panellists mentioned that double-counting may occur if the ‘satisfaction’ that comes with saving (treatment) time may be captured by health-related quality of life measures. Whilst generic preference-based measures of health-related quality of life often account for a reduction in time in illness, it is not clear that these measures are valued in a manner that considers time spent seeking or receiving treatment. Some panellists mentioned that treatment time may be (or partially be) captured by components such as the ‘usual activities’ section of the EuroQol 5-dimensions (EQ-5D) instrument [30] (version not specified), and some child-specific measures of health-related quality of life that contain questions regarding schooling or homework (e.g. the Child Health Utility, nine dimensions (CHU-9D) instrument [31])—something that, as noted by one participant, is “potentially exacerbated if a treatment is ongoing”.

#### Considerations 5 and 6: Whether CYP are Old Enough for Their Time to be Considered Meaningful in Economic Evaluations, and Whether Forgone Time is School or Play Time

Consideration 5, regarding whether CYP are old enough for their time to be considered valuable in the context of an economic evaluation, received the widest spread of ratings for any item. In total, 63.0% of panellists rated this item as 4 or higher (moderately or very important), with over one-third (35.6%) rating it as not important. One panellist (1.4%) did not provide a rating, selecting instead ‘I am not sure or I do not know’. The mean score for this question, excluding this individual, was 4.9, and the median score was 5. Whilst this item was included using our criteria, it reached a low level of agreement—potentially suggesting that it is of limited importance.

General feedback indicated some panellists considered it important to include CYP’s time regardless of their age, whilst others felt it prudent to only include this time once it exhibits a larger opportunity cost (for instance, missed education). This was unsurprising and agreed with the sentiment expressed in an international survey [9], where opinions diverged. There, about a third of all respondents (93/274) said that disregarding CYP’s time in a societal economic evaluation might be justifiable when displaced time relates to very young children, and, in a subsequent question, identified 4–6 years of age as a rough cut-off point, largely on the basis of developmental and educations milestones [9].

This sentiment was further probed by Consideration 6, which asked about whether CYP’s time displaced by treatment is (or would likely be) school or play time. This item also demonstrated a wide range of views. Whilst 72.6% of panellists rated this item as moderately or very important, the mean was close to the centre of the scale, at 5.2, and the median score was 5. According to our pre-specified criteria, this consideration showed a low level of agreement.

Panellists who rated the consideration of whether time displaced is or is likely to be school or play time as moderately or very important were asked to note which types of time they thought a researcher should be inclined to not include in an economic evaluation. A range of views was given by panel members.

The view that all displaced time should be included was the most common sentiment expressed by participants, although opinions varied on whether play time should be valued equally to school time. Some stated that different types of time should be considered equal, expressing views such as “education is important but play-time is also time for children to develop and grow. […] All of this contributes to their ability to optimally develop”. Others gave their opinion that school time ought to be valued higher than play time, and one panellist noted that there exists a ‘friction cost’ to rescheduling time displaced by receiving treatment which “is easier for non-structured time”. Amongst those who expressed views that different types of time might require different valuations, age was frequently discussed—with a common viewpoint that the opportunity cost of missed education grows as a child gets older. Conversely, a few panel members expressed their belief that displaced time “out-of-school hours” or for children “not old enough to be in school” should not be considered in any case. Some panellists suggested more research may be required on the long-term effects of missed play time on the development of CYP.

Some panellists mentioned ethical or moral arguments for including CYP’s time, and for valuing school time and play time equally: “play time is just as important to a young person’s development as formal education”. Certainly, play time has important implications for a child’s development and is in and of itself a learning opportunity, which has significance in terms of life outcomes, and several panellists expressed concern about measuring this time differently. Other panellists stated that not including the time of particularly young CYP would be concerning to them, expressing their belief that this time has an absolute value or is important to include for moral reasons. For instance, one panellist expressed that “formative years may be just as or more so valuable to life outcomes”.

#### Considerations 7 and 8: Whether Including CYPs’ Forgone Time is of Interest to Decision-Makers or Researchers

Almost 96% of panel members rated whether accounting for CYP’s time is likely to be of interest to decision-makers (Consideration 7) as moderately or very important, with a mean score of 6.8 and a median of 7. There was a wide range of scores for this consideration, with 60.3% rating it very important and over one-third of participants rating this item as moderately important.

A similar item referring to whether accounting for CYP’s time is likely to be of interest to the research community (Consideration 8) received slightly less agreement. Some 87.7% of panel members viewed this consideration as moderately or very important, with a mean score of 5.8 and a median of 6. Just over 56% of panellists rated this consideration as moderately important, compared with 31.5% viewing it as very important.

### Differences in Scores Between Rounds

Round 2 was completed by 73 of the panellists who submitted a Round 1 response (81%). For most considerations, changes in scores between rounds were minimal; for five of the eight items, median scores held constant, with mean scores varying by ± 0.2 points or less.

In Round 2, the median score for the consideration of whether the amount of CYP’s time displaced due to receiving treatment is (or is likely to be) substantial enough to warrant inclusion in an economic evaluation fell from 7.5 to 7 (− 0.5 points) in Round 2, with a corresponding reduction in the mean score from 7.1 to 6.8 (− 0.3 points). The two items with the most disagreement in Round 1 also saw the biggest changes in Round 2. Across Rounds, the median score for whether CYP are old enough for their time to be considered valuable within the context of an economic evaluation went down by 1 point to 5, and the mean score reduced by 0.4 points to 4.9. Finally, the median score for the consideration of whether CYP’s time displaced by receiving treatment is (or is likely to be) school or play time was 0.5 points lower in Round 2, and its mean 0.3 lower.

## Discussion

The aim of this study was to produce a list of relevant considerations that can help researchers make decisions about whether and when to include CYP time in their economic evaluations. Panellists’ ratings showed that, when thinking about whether to include displaced CYP time in an economic evaluation, it is very important for researchers to consider whether: (1) inclusion would be in line with specified perspective(s); (2) CYP’s time may already be accounted for in other parts of the evaluation; (3) the amount of forgone time is substantial, either in absolute or relative terms; and (4) inclusion of CYP’s time costs would be of interest to decision-makers. Respondents thought that considerations such as (1) whether inclusion would be of interest to the research community, (2) whether CYP’s time displaced by receiving treatment is ‘school’ or ‘play’ time, and (3) whether CYP’s are old enough for their time to be considered valuable are moderately important.

Answers to follow-up questions provided advice and insights into panellists’ thinking. For example, different ‘criteria’ were offered for judging whether changes in time are substantial enough to warrant inclusion in an economic evaluation, a consideration that both respondents and seminal literature [3, 6] highlight as important. In cases where this consideration is contemplated at the planning stage, before knowing whether an assessed intervention is likely to require substantially more or less of a patient’s time, discussing this with professionals, patients or carers is likely to provide useful guidance.

Several Delphi participants provided general comments expressing their belief that the inclusion of CYP’s time costs may support more patient-centric care, moving away from a strict emphasis on medical costs and outcomes and providing a more holistic view of the costs and benefits, promoting treatments that are more “respectful of children’s time, reducing the burden on them and their families”. Further, some participants highlighted the possibility of interventions aimed at CYP being undervalued in comparison to those targeting adults if CYP’s time is not included in economic evaluations when adults’ time—for which methods of inclusion are readily available—is included.

Unsurprisingly, views diverged on topics that are underpinned by normative questions. Prominent amongst these were questions about the age at which a child’s time exhibits an opportunity cost and should be of interest in an economic evaluation, as well as uncertainties about whether displaced or saved time should be considered more or less valuable depending on whether this is ‘play’ or ‘formal education’ time. Ethical considerations as well as questions about the breadth and aims of economic evaluation (e.g. whether this is to capture changes in the population’s welfare or merely changes in health) inevitably make it difficult to arrive to absolute, universally acceptable answers to such questions. For this, there is a need for the community of health economists (and other stakeholders) to engage in open discussions where diverse viewpoints and perspectives are debated, where possible, on the basis of evidence from empirical research. Further explorations are required here, perhaps involving child psychologists or other specialists who may be able to provide further clarity on the value of time for children of different ages or used for different purposes. Similarly, further work is needed on identifying suitable and dependable approaches for assigning monetary values to children’s time. Various approaches have been suggested in the health economics and economic literature, ranging from use of pragmatic proxy values [32] to stated preference elicitation methods [4], with each presenting different strengths and limitations (see [10] for a review). Encouragingly, several members of this Delphi panel consider answers to such questions to be important and necessary for advancing the methodology of economic evaluation and making these methods applicable to broader populations.

### Strengths and Limitations

This work has several strengths. First, a Delphi design offers notable advantages, which were conducive to the aims of this study. The utilised design allowed an international group of panellists to contribute their views remotely and anonymously, enabling wider participation and limiting acquiescence and interviewer bias [12, 13, 16, 33]. Secondly, the design enables panellists to suggest additional statements to be gathered and presented to the group for subsequent consideration. In line with recommendations, key aims and important design elements of this Delphi exercise—including clear criteria for inclusion and definitions of agreement—were specified in a protocol and agreed upon in advance, before the study was initiated. A detailed protocol that serves as a blueprint for a Delphi study facilitates consistency, reliability and transparency. Secondly, the study’s panel was relatively sizeable, with 73 participants completing both rounds. Indicatively, in a systematic review of Delphi studies, Diamond and colleagues [15] found that nearly 8 out of 10 Delphi studies had fewer than 50 participants in their final round. Panellists were predominantly health economists and noted high self-reported familiarity with the methods associated with conducting economic evaluations of health care interventions, as well as frequent application of these methods in their work. The synthesis of an international panel, consisting of researchers from 16 different countries, allowed for the collection of diverse views, with answers reflecting a range of differing perspectives.

Nonetheless, our study has certain limitations. A constraint of this study, and of Delphi studies in general, is that participation in the panel was determined by self-selection. Due to this, it is difficult to ascertain the reasons for accepting or declining an invitation to contribute to the study. In the present study, panellists were researchers who indicated they would be willing to hear more, and potentially participate in the Delphi, during a previous related survey on the topic [9]. Anonymity arrangements make it impossible to determine whether researchers who opted to be approached about this Delphi were systematically different to researchers who did not, and, similarly amongst those approached, whether those who agreed were dissimilar to those who declined. More fundamentally, without knowing the exact synthesis of the wider population of health economists, it is not possible to know how well the respondents matched this population, or the extent to which the reported views are representative. Secondly, the predetermined criteria for judging the importance of considerations (i.e. the percentage of panellists deeming a consideration as not important/important/very important) may be seen as more inclusive than similar criteria in other studies (see, for instance, [19, 20, 22]). Such criteria are, however, set according to the objectives of each study—in the case of our work, to gain insights into the importance of different key considerations, rather than to produce an exclusive and exhaustive list. Indicatively, adopting stricter criteria (e.g. disregarding all considerations that less than 80% of respondents deem as moderately or very important) would inevitably lead to a narrower selection (in this case, discarding considerations on whether CYP are old enough for their time to be considered valuable, and whether CYP time displaced by receiving treatment is school or play time. Last, it needs to be acknowledged that this survey aimed to gather views of health economists. Anthropologists, developmental psychologists, parents and others may have different views and answers, especially on when CYP’s time is considered ‘valuable’ from their perspective. Rather than gathering views to affirm or counter the (well-established) literature on CYP time use in different activities, and the associated benefits [34, 35], this study set out to record health economists’ views and thoughts on what considerations fellow researchers should take into account when deciding whether to include the opportunity cost of CYP’s time, in the context of health economic evaluations.

## Conclusion

This article presents the findings of a large, international Delphi study that provides a set of guiding considerations which aim to assist researchers when deciding whether to include CYP time-related costs in their economic evaluations. The study also gave an opportunity to gather further insights into these considerations, highlight gaps in our methodological ‘playbook’ and explore normative questions that need to be resolved in the future.

## Supplementary Information

Below is the link to the electronic supplementary material.

Supplementary file1 (PDF 320 kb)

## Declarations

### Availability of data and material

Anonymised data underpinning the reported study can be accessed following a formal request, in accordance with terms and conditions set by the NIHR (funder) and the University of Warwick (contractor).
