# Supplementary material for: Considerations Around the Inclusion of Children and Young People’s Time in Economic Evaluation: Findings from an International Delphi Study
Source: Pharmacoeconomics. 2024 Aug 17;42(11):1267–77. doi: 10.1007/s40273-024-01411-w (PMC11499441; doi:10.1007/s40273-024-01411-w)

Supplementary Materials - PharmacoEconomics

Considerations around the inclusion of Children and Young People's time in economic evaluation: findings from an international Delphi study.

Cameron Morgan[1], Cam Donaldson[2,3], Emily Lancsar[3], Stavros Petrou[4], Lazaros Andronis[1]

[1] Centre for Health Economics at Warwick, University of Warwick, Coventry, UK.
[2] Yunus Centre for Social Business & Health, Glasgow Caledonian University, Glasgow, UK.
[3] Department of Health Services Research and Policy, Australian National University, Canberra, Australia.
[4] Nuffield Department of Primary Care Health Sciences, University of Oxford, Oxford, UK.

Corresponding author: Dr Lazaros Andronis, Centre for Health Economics at Warwick, Warwick Medical School, University of Warwick, Coventry, CV4 7AL. Email: l.andronis@warwick.ac.uk

Table 1. Predetermined study characteristics (adapted from Diamond et al., 2014)

Design aspect	Actions and definitions
**Study objectives**	
What is the aim of this Delphi study?	The planned Delphi study aimed to:<br>• identify a set of items (considerations) that panellists deem to be important (as opposed to not being important) in deciding whether to include CYP time related costs in an economic evaluation.<br>• quantify and present the level of agreement between panellists' responses in relation to the importance of each assessed item.
**Participants**	
How were potential panellists identified? Will panellists be selected or excluded?	Potential panellists were all individuals who expressed an interest in participating in this Delphi study as part of a preceding international survey. All potential panellists were invited to participate.
**Criteria and decisions**	
How will items be selected?	Selection of items after Round 1 responses:<br>• Items that have been scored as 'moderately important' (i.e. 4-6) or 'very important' by at least 60% of all panellists will be selected for inclusion. These items will be forwarded for further consideration in Round 2 of the study.<br>• Items scored as 'not important' region (1-3) by less than 40% of all panellists will be excluded from further consideration.<br><br>Selection of items after Round 2 responses:<br>• Items that have been scored as 'moderately important' (i.e. 4-6) or 'very important' by at least 60% of the panellists will be selected for inclusion. These items will comprise the final list of important considerations.<br>• Items scored as 'not important' region (1-3) by less than 40% of all panellists will be mentioned and discussed but will be excluded from the final list of considerations.
When will new items be included?	New items, suggested directly by panellists in Round 1 will be added to the initial list of considerations and will be presented in Round 2 if they are suggested by at least 10% of respondents.
How will agreement be assessed?	Agreement reached and level of agreement will be assessed at the end of Round 2. This is defined as:

	• Agreement reached; high level of agreement: At least two thirds (≥66.7%) of all panellists have rated an item within the region that the group median lies.<br>• Agreement reached; low level of agreement: Between 33.4% and 66.7% of all panellists have rated an item within the region that the group median lies.<br>• Agreement not reached. Less than 33.4% of all panellists have rated an item within the region that the group median lies.
**Number of rounds**	
What criteria will be used to determine to stop the Delphi process, or will the Delphi be run for a specific number of rounds only?	Given that the primary aim of this Delphi exercise is not to achieve (force) consensus, the study will stop at Round 2. By then, respondents will have had an opportunity to rate items (Round 1), propose new items (Round 1) and re-rate (revise or maintain) their ratings (Round 2).

Table 2. Characteristics of Round 1 and Round 2 panellists

	**Round 1 (n=90)**		**Round 2 (n=73)**	
	**Frequency**	**Percentage[a]**	**Frequency**	**Percentage[a]**

**How would you best describe yourself? (select all that apply)[b]**				
As a health economist	71	78.9%	59	80.8%
As a health care professional	12	13.3%	11	15.1%
As a researcher in a different field or discipline	15	16.7%	10	13.7%
Other	2	2.2%	1	1.4%
**In what capacity do you carry out research in health economics? (select all that apply)[b]**				
As an employee of an academic institution	79	87.8%	63	86.3%
As an employee of a for-profit organisation	7	7.8%	7	9.6%
As an employee of a not-for-profit organisation	7	7.8%	6	8.2%
As a self-employed researcher	5	5.6%	3	4.1%
As a student	3	3.3%	3	4.1%
As an employee of a governmental institution	2	2.2%	2	2.7%
Other	1	1.1%	1	1.4%
**Have you ever been involved in making collective decisions about the allocation of health care resources? (e.g., as part of a funding board, committee, panel etc.)**				
No	50	55.6%	41	56.2%
Yes	38	42.2%	31	42.5%
I don't know or I prefer not to answer	2	2.2%	1	1.4%
**What were these decisions about? (n=38 in Round 1, n=31 in Round 2)**				
Provision of funds for health care research	15	39.5%	12	38.7%
Adoption of health care interventions	10	26.3%	9	29.0%
Both: interventions and research funding	11	28.9%	9	29.0%
Other	2	5.3%	1	3.2%
**In the last five years, how often have you been involved in: Leading or carrying out economic evaluations in health and/or social care?**				
Always or almost always	39	43.3%	30	41.1%
Often	31	34.4%	26	35.6%
Sometimes	14	15.6%	12	16.4%
Rarely	5	5.6%	4	5.5%
Never or almost never	1	1.1%	1	1.4%

**In the last five years, how often have you been involved in: Leading or carrying out research on methodological aspects of economic evaluation in health and/or social care?**				
Always or almost always	20	22.2%	17	23.3%
Often	26	28.9%	22	30.1%
Sometimes	24	26.7%	16	21.9%
Rarely	14	15.6%	12	16.4%
Never or almost never	6	6.7%	6	8.2%
**How familiar would you say you are with the general methodology for conducting economic evaluations in health care?**				
Very familiar	72	80.0%	58	79.5%
Familiar	14	15.6%	12	16.4%
Somewhat familiar	4	4.4%	3	4.1%
Unfamiliar	0	0.0%	0	0.0%
**How familiar would you say you are with the categories of costs and outcomes that should typically be included in analyses carried out from different perspectives (e.g., patient, health care system, societal, etc.)**				
Very familiar	76	84.4%	60	82.2%
Familiar	13	14.4%	12	16.4%
Somewhat familiar	1	1.1%	1	1.4%
Unfamiliar	0	0.0%	0	0.0%
**How familiar would you say you are with the measurement, valuation and inclusion of the opportunity cost of individuals' time in economic evaluation.**				
Very familiar	39	43.3%	31	42.5%
Familiar	40	44.4%	33	45.2%
Somewhat familiar	8	8.9%	6	8.2%
Unfamiliar	3	3.3%	3	4.1%
**In which country are you based?**				
United Kingdom	34	37.8%	29	39.7%
United States	17	18.9%	11	15.1%
Australia	12	13.3%	10	13.7%
Canada	10	11.1%	8	11.0%
Sweden	3	3.3%	3	4.1%

Germany	2	2.2%	2	2.7%
Netherlands	2	2.2%	1	1.4%
Costa Rica	1	1.1%	1	1.4%
Brazil	1	1.1%	1	1.4%
India	1	1.1%	1	1.4%
Mexico	1	1.1%	1	1.4%
Japan	1	1.1%	1	1.4%
Italy	1	1.1%	1	1.4%
South Africa	1	1.1%	0	0.0%
Colombia	1	1.1%	1	1.4%
Uzbekistan and UK	1	1.1%	1	1.4%
US and Australia	1	1.1%	1	1.4%

[a] Percentage of participants in each round who selected each option (n=90 in Round 1, n=73 in Round 2).

[b] Percentages for these questions may exceed 100, as participants were able to select more than one answer.

Fig 2. Percentage of panellist ratings received in each category during Round 2.

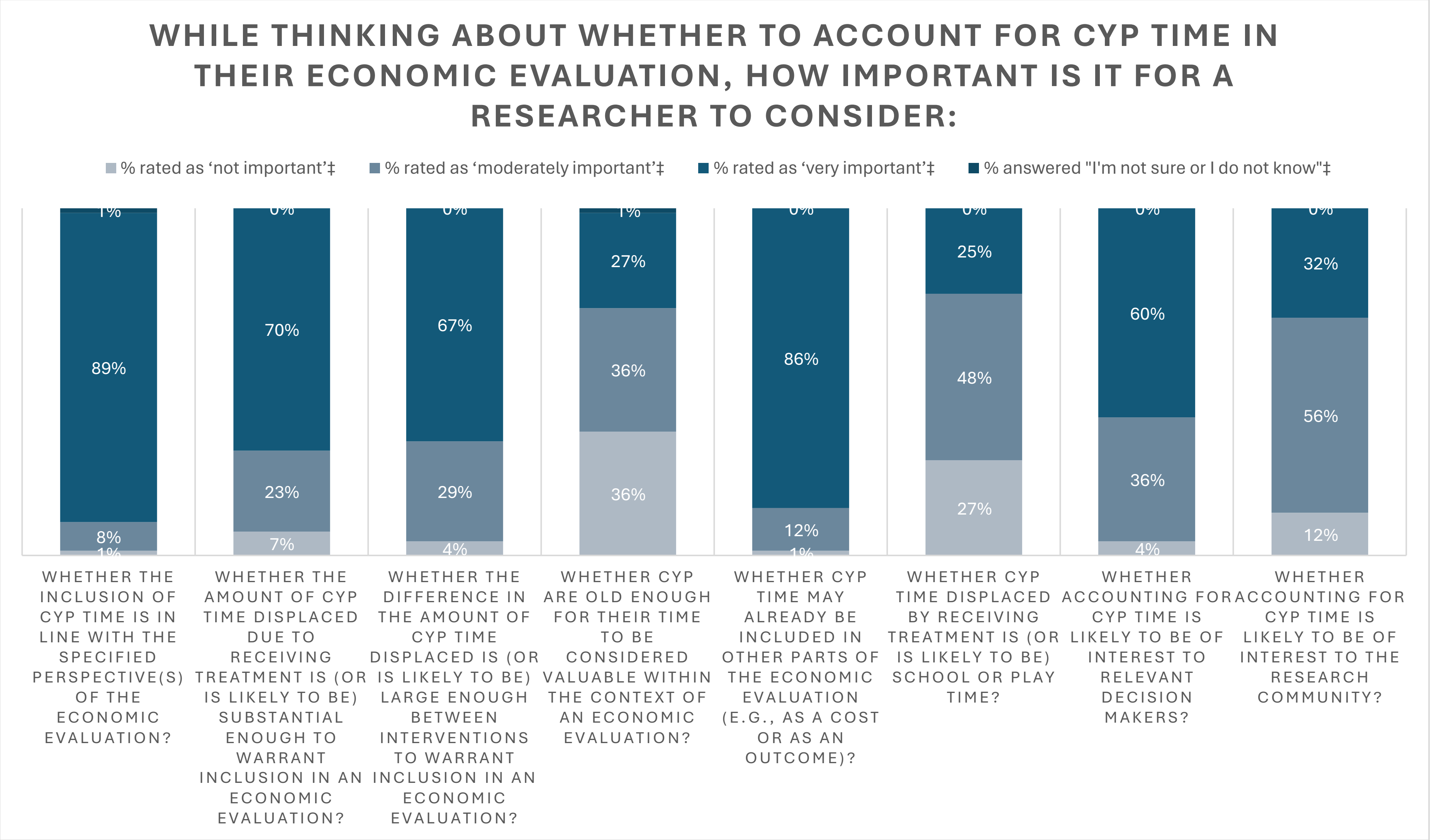

Supplement: Supplementary file 1 — Supplementary file1 (PDF 320 kb) [file 40273_2024_1411_MOESM1_ESM.pdf]
